# Information seeking of French parents regarding infant and young child feeding: practices, needs and determinants

**DOI:** 10.1017/S1368980021003086

**Published:** 2022-04

**Authors:** Sofia De Rosso, Sophie Nicklaus, Pauline Ducrot, Camille Schwartz

**Affiliations:** 1 Centre des Sciences du Goût et de l’Alimentation, AgroSup Dijon, CNRS, INRAE, Université Bourgogne Franche-Comté, F-21000 Dijon, France; 2 Santé Publique France, French National Public Health Agency, F-94415 Saint-Maurice, France

**Keywords:** Child feeding guidelines, Infant feeding, Parents’ information sources, Public health communication, Complementary feeding information

## Abstract

**Objective:**

As part of an update of feeding benchmarks targeting children aged 0–3 years, this study aimed to explore parental perceptions, information-seeking practices and needs concerning infant and young child feeding (IYCF) to design an efficient communication strategy.

**Design:**

Participants were recruited using the quota sampling to complete an online survey. Effects of parity, child age, prematurity, parental education and financial situation on parents’ responses were evaluated separately.

**Setting:**

France.

**Participants:**

A nationally representative sample of 1001 parents of children <4 years.

**Results:**

Parents whose child had any medical condition affecting feeding (children with medical condition (CMC), 17 %) were considered separately from healthy children’s parents. All the healthy children’s parents recognised the importance of IYCF for children’s health and growth; however, one-third considered the available advice contradictory and not guilt-free. The most used information sources were healthcare professionals (HCP, 81 %), internet (72 %) and parental networks (63 %). The most influential sources (mean influence ± sd) included HCP (7·7 ± 1·7/10), childcare professionals (7·3 ± 1·8/10) and parental networks (6·9 ± 1·8/10). Parents searched for practical tips for implementing IYCF starting when their child was 5 months old. Differences regarding the type of source used by parents with higher *v*. lower educations were small. Search strategies differed according to parity or child age but not to prematurity. The CMC parents reported slightly different practices and needs.

**Conclusions:**

Parents receive information from multiple sources, which can lead to confusion when deciding which advice to follow. A public health communication strategy adapted to the current parental needs should target these various sources.

During the first 1000 d of the life of a child, individual dietary patterns develop rapidly, and they can impact risk factors for later-life obesity and health in general. This period constitutes a perfect window of opportunity to implement health-related programmes that promote healthy eating and growth in young children^(1–3)^. Children’s eating behaviours (e.g. food preferences and appetite control abilities) are also established at this stage, particularly during complementary feeding (CoF)^(4–6)^. CoF is the period in the life of a child when foods other than milk start to be introduced into the diet^(7)^; this is a dietary transition that typically covers the first years of life. During this phase, parental influence on feeding practices is essential in shaping infant eating behaviours^(8)^.

Previous studies have explored parental behaviours and feelings regarding infant and young child feeding (IYCF), and they demonstrated that most parents are aware of the importance of their role in shaping the early foundations of their children’s diet^(9,10)^. However, for parents, especially mothers, the pressure of ‘doing well’ and the feeling of being responsible for the health of their child can easily turn into a source of stress, which may lead them to feel strained in their parental role^(11–13)^. Parents look for information and experience-related feedback regarding the IYCF process. However, they often feel impotent and frustrated when receiving official recommendations, because they find them too demanding^(14)^.

The social ecological model has been used frequently to develop frameworks for prevention; it explains the theory about how the behaviour of an individual is influenced by multiple factors^(15)^. This approach applies to parental feeding behaviour, which is influenced by many interacting components, both intrinsic (gender, age) and environmental (family, community and society)^(15,16)^. Increasing the IYCF literacy of parents and empowering them can foster a change towards healthier feeding practices. However, as shown in social and behavioural change communication theory, interactive approaches and mixed communication strategies are required to reach these goals^(17)^. Parents can rely on different sources to gather information and advice on IYCF, including previous experience and professional (paediatricians, nurses, midwives and general practitioners) and nonprofessional (family, friends, online forums and blogs) sources^(18,19)^, and not forgetting the influence of social media, which has recently been successfully tested as an effective means to spread IYCF-related information^(20)^.

The source of information used by mothers changes as their child grows^(21)^. A French national survey conducted in 2013 suggested that primiparous mothers with infants < 12 months old are more likely to seek advice from HCP than multiparous mothers^(19)^. It is therefore topical to explore which information sources parents currently use and the influence these sources have on parental IYCF decisions. Education is also linked to the forms of advice sought by parents. According to a study set in five European countries (England, Finland, Germany, Hungary and Spain), mothers who have received education beyond the age 16 years reportedly rely more on written sources, HCP and family/friends^(22)^. A qualitative and quantitative study conducted in a socially deprived area in Scotland reported that the primary sources of CoF information are family/friends (91 %), the internet (89 %) and health visitors (77 %)^(23)^. Although many public health authorities issue IYCF recommendations, related adherence is often low^(24,25)^. More insights are needed regarding parents’ perception of IYCF information (accessibility and understanding) to facilitate compliance with official recommendations.

Parents of premature children might rely even more on medical advice than on other sources, but this reliance has not been extensively documented. Notably, parents of premature children look for information more actively than do other parents^(26)^. However, no official documents specifically address premature child feeding. For the parents of premature children, the absence of specific guidance about child feeding could contribute to feeling uncertain and lacking confidence about feeding practices, thereby accentuating parental frustration. The same might also apply to parents of children with medical conditions that could impact their diet (e.g. cow’s milk protein allergy or nasogastric intubation), but it has not been extensively studied. Considering their specific or similar information-seeking practices compared with those of parents of healthy children may allow for the optimisation of resources in regard to producing public health information material.

The literature related to information source use by parents to obtain IYCF information is not extensive. There has been a limited exploration of how much those sources are considered influential in determining parental decisions in IYCF, especially from a quantitative point of view with a nationally representative sample and regarding the changing landscape of information and communications technology in public health and prevention. In France, the former official IYCF recommendations are not recent (the related communication material was released in 2004), but new IYCF recommendations for children aged 0–3 years old have recently been published^(27)^. In this context, to prepare a public health communication strategy to spread those recommendations to the lay public, it is particularly necessary to focus on the information-seeking practices of parents with different profiles.

Therefore, the aim of the present study is first to describe the perceptions of French parents of children < 4 years old about IYCF (e.g. is it important for the health and growth of the child? Has it been a source of concern? Is it easy to find information on IYCF?), how informed they feel and whether these perceptions differ according to selected socio-demographic characteristics (parental education, perceived financial situation, parity, child age and prematurity). The second aim is to explore parental practices related to searches for guidance about IYCF (e.g. when do they look for information, what type of content do they look for, which sources of information do they use and how much are they influenced by those sources) and whether these practices differ according to the abovementioned socio-demographic characteristics. The final objective is to explore the same topics among parents of children with a medical condition that could have an impact on their diet in comparison with parents of healthy children.

## Methods

### Study design, setting and sampling procedure

The present descriptive study was conducted in France in the form of an online survey that was open for completion from the 10th to the 29th of January 2020. The recruitment procedure was managed by a private research and consulting firm, by applying the quota sampling method. This method is a non-probabilistic sampling approach that employs purposeful selection criteria to include the participants. It allows researchers to study a characteristic of a particular subgroup of the population (in our case, parents of children aged below 4 years) by giving an accurate representation of the population of interest. The quota method was applied to the following variables: age of the parent, profession of the household reference person (defined as the person earning the most in the household), region, living area (rural, urban) and first-time or multiparous parent. The sample was drawn from the panel of eligible respondents to the research and consulting firm, and it was made to ensure national representativeness by calibrating the participant data according to the general population census^(28)^. We targeted a representative sample of 1000 French parents of children < 4 years old. Those 1000 parents were the first respondents presenting the required characteristics to answer the questionnaire. The sample size of 1000 was defined a priori, and it was considered sufficient to represent our population of interest according to previous surveys conducted by the private research and consulting firm with the same sampling method on similar subgroups of the population.

### Questionnaire

A full description of the information collected using the questionnaire is listed in online supplementary material, Supplemental Material 1, and the original version of the questionnaire (in French) is presented in online supplementary material, Supplemental Material 2. The questions and the list of multiple choice answers were developed based on previous studies^(12,19)^. The questionnaire was divided into five sections: characteristics of the youngest child, the parents and the household; parents’ perceptions of IYCF; parents’ perceptions of IYCF information; the sources of information used by parents with relative influence of the chosen sources on their IYCF practices; and the type of information (content) sought by parents. All the parents were asked to refer to their youngest child when answering the questionnaire. Multiparous parents were asked to answer referring their youngest child, except one question asked only to multiparous parents (‘CoF is easier for my last child than for the first one(s)’). The survey included closed-ended multiple choice questions, with one or check-all-that-apply answer options, as well as questions for which the respondents rated their answer on a scale. A continuous scale was used to evaluate how much the parents were influenced by a chosen source of information in making IYCF-related decisions (from 1 to 10, with 1 meaning the source did not influence their decisions at all and 10 meaning it influenced their decisions very strongly). A 4-point Likert scale (strongly agree, tend to agree, tend to disagree and strongly disagree) was used to ask the parents their degree of agreement about some statements regarding perceptions and feelings around IYCF. The questionnaire was reviewed by public health experts and by the private research and consulting firm in charge of the recruitment process.

### Statistical analysis

For all the statistical analyses, R version 3.6.1 was used^(29)^. Following descriptive data analysis, parents whose the youngest child has or had a medical condition that could have a serious impact on his/her diet (CMC) were treated as a separate subsample from parents with healthy children. Based on the assumption of potentially different information-seeking behaviour, analyses were then run separately for the two subsamples of parents. Both subsamples were representative of the study population. Two-way *χ* ^2^ tests of independence were calculated to evaluate whether having medical conditions that could affect the diet was associated with any socio-demographic characteristic.

Frequencies, percentages and means ± sd are used to describe the results. Statements requiring an answer on a 4-point Likert scale were considered discrete and were dichotomised as ‘agree’ or ‘disagree’ for the analysis. Where appropriate, the *χ* ^2^ was used to determine whether the relationship between two variables was statistically significant. In the case of a quasi-continuous variable (i.e. the degree of influence of used sources of information, scale from 1 to 10), one-way ANOVA or independent two-sample *t* tests were used to compare the means of the different groups. Two-way *χ* ^2^tests of independence were calculated to compare the results of parents of healthy children *v*. CMC (for this subsample, detailed results are presented in online supplementary material, Supplemental Material 3). Assumptions of normality for each test were checked and met. Significance was set at *P* < 0·05.

## Results

### Participants

The two subsamples of the study population are described in Table 1. Answers were obtained from a total of 1001 parents who were representative of French parents with at least one child < 4 years old. Among them, 175 reported having CMC (medical conditions: gastroesophageal reflux disease, cow’s milk protein allergy, nasogastric intubation or congenital abnormalities of the digestive tract) and were excluded from the primary analysis. The subsample of the remaining 826 parents is also representative of the French population, because no differences were observed from the original study population regarding the quota variables (Table 1). In other respects, some significant differences were found between the two subsamples, with a higher percentage of premature children (17 % *v*. 10 %, respectively; *P* = 0·01) and more fathers than mothers answering the survey (26 % *v*. 18 %, respectively; *P* = 0·02) in the CMC subsample.

**Table 1:** Characteristics of the study sample (*n* 1001) and the two subsamples: parents of healthy children (*n* 826) and parents of children with a medical condition that could affect their diet (*n* 175)*

Characteristics	All		HC		CMC		*P* †
	*n*	%	*n*	%	*n*	%	
All	1001		826	83	175	17	
Child characteristics							
Age of the youngest child							0·59
< 12 months	277	28	225	27	52	30	
12–23 months	260	26	217	26	43	25	
24–35 months	260	26	220	27	40	22	
≥ 36 months	204	20	164	20	40	23	
Gender							0·15
Female	510	51	430	52	80	46	
Male	491	49	396	48	95	54	
Born preterm							**0·01**
Yes	110	11	81	10	29	17	
No	891	89	745	90	146	83	
Eating food other than milk							0·80
Yes	889	89	735	89	154	88	
No	112	11	91	11	21	12	
Responding parents’ characteristics							
Gender							**0·02**
Female	803	80	674	82	129	74	
Male	198	20	152	18	46	26	
Age							0·13
Less than 35 years old	604	60	489	59	115	66	
35 years old and more	397	40	337	41	60	44	
Education level (years)							0·18
< A level	179	18	141	17	38	22	
≥ A level	822	82	685	83	137	88	
Socio-professional category of the interviewed parent							0·47
High	371	37	308	37	63	36	
Low	467	47	379	46	88	50	
No occupation/retired	163	16	139	17	24	14	
Mother tongue							0·26
French	942	94	781	95	161	92	
Other	59	6	45	5	14	8	
Parity							1
Primiparous	388	39	320	39	68	39	
Multiparous	613	61	506	61	107	61	
Household characteristics							
Self-perception of financial situation							0·59
Good	457	46	383	46	74	42	
Difficult	535	53	436	53	99	57	
No answer	9	1	7	1	2	1	
Living area							0·18
Rural	432	43	360	44	72	41	
Urban	569	57	466	56	103	59	
Socio-professional category of the household reference parent‡							0·08
High	456	46	383	46	73	42	
Low	501	50	412	50	89	51	
No occupation/retired	44	4	31	4	13	7	

HC, parents of healthy children; CMC, parents of children with medical condition that could affect the diet.

* For results regarding parents of CMC, refer also to supplementary material, Supplemental Material 3.

† Based on *χ* ^2^ tests, comparing parents of HC and parents of CMC. Significant *P*-values are in boldface.

‡ The parent with the highest income.

### Parent perceptions of infant and young child feeding (including complementary feeding) and how informed they feel

Among parents of healthy children (*n* 826), 98 % were aware of the importance of IYCF for the current and future health and growth of their child (Table 2). In addition, 99 % of parents were aware that this period is important for acquiring healthy eating habits. Overall, the CoF period was experienced fairly well by parents, with 92 % considering that it went well. However, for 30 % of the parents, the CoF turned out to be a source of concern. Being a multiparous parent did not necessarily facilitate CoF with the younger child, with only 53 % considering that CoF was easier for the younger child than for the older ones. Eighty-eight percentage of the parents considered themselves well-informed, and 86 % were satisfied with the available information. Eighty-eight percentage of the parents also considered finding information about IYCF to be easy to understand and reported that the available information answered their questions. Thirty-two percentage of the parents indicated that the available information could be contradictory, and 30 % reported that the information gave the perception that they were not implementing IYCF correctly.

**Table 2:** Perceptions (frequencies and percentages of parents who answered positively for each item) of IYCF (including CoF) and of IYCF information of parents of healthy children (*n* 826), comparisons according to selected socio-demographic characteristics

			Characteristics																							
			Self-perception of financial situation (*n* 819)†				Education				Parity status				Age of the youngest child (months)								Born preterm			
	Total		Good		Difficult		< A level		≥ A level		Primiparous		Multiparous		< 12		23-Dec		24–35		≥ 36		Yes		No	
	*n*	%	*n*	%	*n*	%	*n*	%	*n*	%	*n*	%	*n*	%	*n*	5	*n*	%	*n*	%	*n*	%	*n*	%	*n*	%
Total	826		383		436		141		685		320		506		225		217		220		164		81		745	
Perceptions of IYCF (including CoF)																										
CoF for my youngest child is going well or it went well	**762**	**92**	351	92	404	93	134	95	628	92	301	94	461	91	202	90	195	90	204	93	161	98	74	91	688	92
*P* *			0·59				0·17				0·12				**0·01**								0·75			
It is easy to find information on IYCF (including CoF)	**727**	**88**	339	88	382	88	129	92	598	87	274	86	453	90	198	88	192	89	190	86	147	90	72	89	655	88
*P* *			0·69				0·16				0·09				0·8								0·8			
CoF is easier for my last child than for the first one (only multiparous, *n* 506)	**269**	**53**	122	55	145	52	49	50	220	54					65	46	71	56	78	59	55	51	31	54	238	53
*P* *			0·57				0·48								0·18								0·84			
CoF for my last child is or it has been source of concern	**252**	**30**	110	30	141	32	49	35	203	30	127	40	125	25	76	34	63	29	71	32	42	26	25	31	227	31
*P* *			0·34				0·23				**< 0·001**				0·31								0·94			
IYCF is important for the present and future health of my child and for his growth	**815**	**98**	375	98	433	99	136	97	679	99	315	98	500	99	219	97	217	100	216	98	163	99	80	99	735	99
*P* *			0·08				**0·01**				0·64				0·07								0·93			
IYCF is important for the establishment of good eating habits	**819**	**99**	378	99	434	99	138	98	681	99	317	99	502	99	222	99	216	99	219	99	162	99	80	99	739	99
*P* *			0·19				0·07				0·82				0·64								0·69			
Perceptions of IYCF information																										
I feel well informed about IYCF	**726**	**88**	337	88	383	88	126	89	600	88	267	83	459	91	183	81	193	89	199	91	151	92	68	84	658	88
*P* *			0·95				0·56				**0·002**				**0·004**								0·25			
I am satisfied with the available information on IYCF (including CoF)	**714**	**86**	332	87	376	86	123	87	591	86	261	82	453	90	185	82	186	86	190	86	153	93	67	83	647	87
*P* *			0·85				0·76				**0·001**				**0·02**								0·3			
The available information on IYCF (including CoF) answers to my questions	**723**	**88**	335	88	382	88	129	92	594	87	274	86	449	89	188	84	193	89	191	87	151	92	69	85	654	88
*P* *			0·95				0·12				0·19				0·08								0·5			
The available information on IYCF (including CoF) is clear, easy to understand	**744**	**90**	340	89	397	91	132	94	612	89	283	88	461	91	198	88	194	89	201	91	151	92	72	89	672	90
*P* *			0·28				0·12				0·21				0·51								0·71			
The available information on IYCF (including CoF) is easy to put into practice	**710**	**86**	336	88	369	85	125	89	585	85	267	83	443	88	197	88	183	84	184	84	146	89	65	80	645	87
*P* *			0·2				0·31				0·1				0·36								0·12			
The available information on IYCF (including CoF) is contradictory	**260**	**32**	118	31	139	32	49	35	211	31	114	36	146	29	74	33	71	33	68	31	47	29	28	35	232	31
*P* *			0·74				0·36				**0·04**				0·8								0·53			
The available information on IYCF (including CoF) is giving me the perception of not implementing correctly IYCF	**251**	**30**	109	29	138	32	40	28	211	31	96	30	155	31	52	23	66	30	79	36	54	33	25	31	226	30
*P* *			0·32				0·57				0·85				**0·03**								0·92			

IYCF, infant and young child feeding; CoF, complementary feeding.

This table shows frequencies and percentages of parents, with the correspondent characteristics, that answered positively (strongly agree and tend to agree) to the statements in the left column. The second column shows the total of parents that answered positively (strongly agree and tend to agree) on the statements in the left column.

* Based on *χ* ^2^ tests, comparing perceptions and feelings on IYCF with selected characteristics of parents (*n* 826). Significant *P*-values are in boldface.

† *n* 7 parents did not give information on their financial situation and were excluded from the analysis.

A higher proportion of parents of 3-year-olds found that CoF went well (98 %) compared with parents of younger children (1- to 2-year-old children, 90 %; *P* = 0·01). Parents of younger children (< 12 months) were less satisfied with the available information on IYCF (82 % *v*. 93 % for parents of 3-year-old children; *P* = 0·02) and felt less well informed about it (81 % *v*. 92 % for parents of 3-year-old children; *P* = 0·004). A smaller proportion of parents of younger children (< 12 months) stated that the available information on IYCF gave them the perception that they were not implementing IYCF correctly (23 % *v*. 33 % for parents of 3-year-old children; *P* = 0·03) (Table 2). Primiparous parents reported that the progression of CoF was more a cause of concern than it was for multiparous parents (40 % *v*. 25 %; *P* < 0·001); they also felt less informed (83 % *v*. 91 %; *P* = 0·002) and less satisfied with the available information (82 % *v*. 90 %; *P* = 0·001). Primiparous parents found that the information was contradictory more frequently than multiparous parents (36 % *v*. 29 %; *P* = 0·04).

### Parental practices related to searches on guidance about infant and young child feeding (including complementary feeding)

#### When parents look for information on infant and young child feeding (including complementary feeding) and related content, what do they look for?

Seventy-nine percentage of parents looked for information on IYCF for at least one of their children; 72 % did so for their youngest child. The mean age of the child when the parents began to look for information specifically on CoF was 4·6 ± 3·6 months (median = 4 months; IQR 3–6 months). Seventy-two percentage of the parents reported that they were advised by healthcare professionals (HCP) during regular follow-up consultations. The subject of IYCF, including CoF, was either spontaneously broached by HCP or as a result of specific questions from parents; in total, 80 % of the parents received advice during consultations. Regarding the search content, the parents’ searches were focused primarily on examples of recipes/menus (63 %), portion sizes for complementary foods and milk (55 %), age and modalities of the introduction of foods (54 %) and feeding strategies to address children’s specific behaviours (53 %), for example, how to offer food in case of food refusal or how to address ‘small’ or ‘big’ appetites (Table 3).

**Table 3:** Type of content that parents of healthy children (*n* 826) look for: comparison according to selected socio-demographic characteristics (frequencies and percentages of parents who selected each item)

		Characteristics																								
			Self-perception of financial situation (*n* 819)†				Education				Parity status				Age of the youngest child (months)								Born preterm			
	Total		Good		Difficult		< A level		≥ A level		Primiparous		Multiparous		< 12		12–23		24–35		≥ 36		Yes		No	
	*n*	%	*n*	%	*n*	%	*n*	%	*n*	%	*n*	%	*n*	%	*n*	%	*n*	*%*	*n*	%	*n*	%	*n*	%	*n*	%
Totals	**826**		**383**		**436**		**141**		**685**		**320**		**506**		**225**		**217**		**220**		**164**		**81**		**745**	
Subtotal ‘Menus, recipes’	**523**	**63**	223	58	296	68	92	65	431	63	204	64	319	63	135	60	147	68	135	61	106	65	48	59	475	64
*P* *			**0·004**				0·6				0·84				0·34								0·42			
Example of menus	348	42	147	38	199	46	61	43	287	42	135	42	213	42	84	37	96	44	96	44	72	44	33	41	315	42
*P* *			**0·04**				0·77				0·98				0·4								0·79			
Example of recipes	389	47**)**	160	42	225	52	74	53	315	46	154	48	235	46	103	46	107	49	98	45	81	49	33	41	356	48
*P* *			**0·005**				0·16				0·64				0·68								0·23			
Subtotal ‘Portion sizes’	**452**	**55**	218	57	230	53	57	40	395	58	183	57	269	53	136	60	110	51	118	54	88	54	45	56	407	55
*P* *			0·23				**< 0·001**				0·26				0·21								0·87			
Portions sizes of food	372	45	172	45	197	45	46	33	326	48	148	46	224	44	101	45	93	43	105	48	73	45	38	47	334	45
*P* *			0·94				**0·001**				0·58				0·78								0·72			
How to adapt milk quantities when introducing other foods	175	21	92	24	81	19	22	16	153	22	80	25	95	19	73	32	38	18	34	16	30	18	18	22	157	21
*P* *			0·06				0·08				**0·03**				**< 0·001**								0·81			
Subtotal ‘Age and modalities of introduction’	**442**	**54**	221	58	217	50	73	52	369	54	186	58	256	51	151	67	106	49	97	44	88	54	47	58	395	53
*P* *			**0·02**				0·65				**0·03**				**< 0·001**								0·39			
Different food groups	275	33	134	35	138	32	48	34	227	33	110	34	165	33	93	41	71	33	49	22	62	38	29	36	246	33
*P* *			0·31				0·84				0·6				**< 0·001**								0·61			
First food pieces	205	25	105	27	97	22	29	21	176	26	87	27	118	23	90	40	44	20	40	18	31	19	17	21	188	25
*P* *			0·09				0·2				0·21				**< 0·001**								0·4			
First foods other than milk	192	23	97	25	94	22	36	26	156	23	75	23	117	23	67	30	42	19	47	21	36	22	19	24	173	23
*P* *			0·2				0·48				0·91				0·05								0·96			
Subtotal ‘Feeding strategies’	**435**	**53**	191	50	242	56	59	42	376	55	164	51	271	54	97	43	123	57	124	56	91	56	46	57	389	52
*P* *			0·11				**0·005**				0·52				**0·01**								0·43			
How to present food in case of refusal	294	36	130	34	162	37	37	26	257	38	113	35	181	36	67	30	81	37	85	39	61	37	29	36	265	36
*P* *			0·34				**0·01**				0·89				0·2								0·97			
How to deal with ‘little’ appetite	188	23	83	22	104	24	28	20	160	23	75	23	113	22	33	15	63	29	55	25	37	23	13	16	175	24
*P* *			0·46				0·37				0·71				**0·003**								0·13			
How to deal with ‘big’ appetite	108	13	42	11	66	15	14	10	94	14	42	13	66	13	32	14	35	16	24	11	17	10	16	20	92	12
*P* *			0·08				0·22				0·97				0·26								0·06			
‘How to feed a child to promote the development of healthy eating habits’	**258**	**31**	136	36	120	28	34	24	224	33	110	34	148	29	73	32	69	32	73	33	43	26	25	31	233	31
*P* *			**0·01**				**0·05**				0·12				0·47								0·94			
‘How to interpret the child’s hunger and satiety cues’	**207**	**25**	103	27	103	24	33	23	174	25	89	28	118	23	47	21	59	27	56	26	45	27	23	28	184	25
*P* *			0·28				0·62				0·15				0·37								0·47			

The second column shows the total of parents who selected the item in the first column. The ‘Subtotals’ shows the number of parents who selected at least one of the items related to that topic.

† *n* 7 parents did not give information on their financial situation and were excluded from the analysis.

* Based on *χ* ^2^ tests, comparing the content that parents look for with selected characteristics of them. Significant *P*-values are in boldface.

A higher proportion of parents with self-perceived difficult financial situations looked more frequently for examples of menus/recipes (68 % *v*. 58 %; *P* = 0·004) and less frequently for information about age and modalities for the introduction of foods (50 % *v*. 58 %; *P* = 0·02) or how to feed a child to promote the development of healthy eating habits (28 % *v*. 36 %; *P* = 0·01). Parents with more years of formal education in comparison with parents with fewer years of formal education were more frequently interested in feeding strategies (55 % *v*. 42 %; *P* = 0·005) and in the definition of portion sizes for complementary foods (58 % *v*. 40 %; *P* < 0·001). They were also more frequently interested in how to feed a child to promote the development of healthy eating habits (33 % *v*. 24 %; *P* = 0·05). The topics related to the age and modalities of the introduction of foods were more relevant for primiparous than multiparous parents (58 % *v*. 51 %; *P* = 0·03) and for parents of children < 1-year-old (67 % *v*. 44 % for 2-year-olds; *P* < 0·001). Parents with children > 1 year were more interested in feeding strategies (57 % *v*. 43 % for < 12 months; *P* = 0·01). Premature birth was not associated with specific information searches.

#### Sources of information used by parents and their degree of influence

As shown in Table 4, HCP were the primary source of information for 81 % of parents, and their level of influence was strong (7·7 ± 1·7/10). Among HCP, paediatricians and general practitioners were the primary vectors of information (each used by 47 % of parents). The internet (including websites, blogs, social networks, applications for smartphones) was the second source of information, being consulted by 72 % of the parents; however, its influence was low (5·6 ± 2·1). Parental networks were a source of information for 63 % of the parents, and their influence was quite high (6·9 ± 1·8). Paper-based tools (e.g. books, newspapers) were a source of information for 44 % of the parents, and their influence was slightly lower (6·2 ± 1·8). Although childcare professionals had a strong influence (7·3 ± 1·8), nearly as strong as that of HCP, they were among the sources that parents used the least (30 %). Only 24 % of the parents used media (radio, television), and they were weakly influenced by these sources (5·8 ± 2).

**Table 4:** Utilisation (frequencies and percentages) and influence (means ± sd) of the different sources of information on IYCF of parents of healthy children (*n* 826), comparisons according to selected socio-demographic characteristics

	Sources of information on IYCF																							
	HCP				Internet				Parents’ network				Paper				Childcare professionals				Media			
	Use†		Influence‡		Use†		Influence‡		Use†		Influence‡		Use†		Influence‡		Use†		Influence‡		Use†		Influence‡	
	*n*	%	Mean	sd	*n*	%	Mean	sd	*n*	%	Mean	sd	*n*	%	Mean	sd	*n*	%	Mean	sd	*n*	%	Mean	sd
Parents *n* 826	669	81	7·7	1·7	594	72	5·6	2·1	517	63	6·9	1·8	360	44	6·2	1·8	251	30	7·3	1·8	197	24	5·8	2·0
Child characteristic																								
Age of the youngest child																								
< 12 months	186	83	7·9	1·5	175	78	6·0	2·0	128	57	7·0	1·7	103	46	6·1	1·8	60	27	7·2	2·0	55	24	5·8	1·9
12–23 months	181	83	7·8	1·6	164	76	5·7	2·0	139	64	6·7	1·8	89	41	6·1	1·7	64	30	7·6	1·4	39	18	5·7	2·3
24–35 months	171	78	7·4	1·9	154	70	5·2	2·2	143	65	6·8	1·9	99	45	6·2	1·8	77	35	7·2	1·8	56	26	5·6	1·9
≥ 36 months	131	80	7·5	1·6	101	62	5·7	2·0	107	65	7·0	1·5	69	42	6·4	1·8	50	31	7·5	2·0	47	29	6·2	2·0
*P* *	0·41		**0·04**		**0·002**		**0·01**		0·22		0·23		0·72		0·57		0·29		0·47		0·09		0·61	
Born preterm																								
Yes	63	78	7·6	1·8	55	68	5·7	2·2	56	69	7·1	1·7	34	42	6·4	1·6	27	33	8·1	1·4	18	22	6·1	2·2
No	606	81	7·7	1·6	539	72	5·6	2·0	461	62	6·8	1·8	326	44	6·2	1·8	224	30	7·3	1·9	179	24	5·8	1·9
*P* *	0·53		0·87		0·47		0·94		0·25		0·36		0·76		0·56		0·54		**0·03**		0·72		0·52	
Parental characteristic																								
Self-perceived financial situation (*n* 819)§																								
Good	311	81	7·9	1·5	283	74	5·7	2·0	239	62	6·9	1·7	174	45	6·3	1·7	119	31	7·6	1·8	93	24	6·0	1·7
Difficult	353	81	7·7	1·7	306	70	5·6	2·1	273	63	6·9	1·8	181	41	6·2	1·8	130	30	7·4	1·8	103	24	5·8	2·0
*P* *	0·93		**0·05**		0·24		0·86		0·95		0·67		0·26		0·43		0·7		0·2		0·83		0·6	
Education																								
< A level	119	84	7·1	2·0	100	71	5·4	2·4	99	70	6·5	2·2	64	45	5·8	1·9	51	36	7·2	1·7	46	33	6·0	2·4
≥ A level	550	80	7·8	1·5	494	72	5·7	2·0	418	61	7·0	1·6	296	43	6·3	1·7	200	29	7·4	1·8	151	22	5·8	1·8
*P* *	0·25		**0·001**		0·77		0·16		**0·04**		**0·01**		0·63		**0·03**		0·1		0·52		**0·007**		0·5	
Parity status																								
Primiparous	268	84	7·8	1·6	259	81	5·8	2·0	239	75	6·9	1·7	152	48	6·3	1·8	107	33	7·6	1·8	74	23	6·0	1·8
Multiparous	401	79	7·6	1·7	335	66	5·5	2·1	278	55	6·8	1·8	208	41	6·1	1·8	144	29	7·2	1·8	123	24	5·8	2·1
*P* *	0·11		0·08		**< 0·001**		0·09		**< 0·001**		0·6		0·07		0·29		0·13		0·06		0·7		0·51	

IYCF, infant and young child feeding; HCP, healthcare professionals.

The columns about the use of the sources show frequencies and percentages of parents who selected that source. The columns about the influence show the mean (±sd) of the sources (parents rated sources on a scale from 1 to 10 and this variable was considered as continuous).

* Based on *t* tests to compare means of two subgroups, ANOVA to compare the means of more than two subgroups; if ANOVA was significant, then specific comparisons were performed with post-hoc tests. Significant *P*-values are in boldface.

† *n* (%).

‡ Mean ± sd.

§ *n* 7 parents did not give information on their financial situation and were excluded from the analysis.

Although some differences were significant with regard to the levels of influence of the sources according to parental characteristics, their amplitude was rather small (< 1 point of difference, Table 4). Parents of children < 1-year-old used the internet more frequently (78 % *v*. 62 % for parents of 3-year-olds; *P* = 0·002). Parental networks were more frequently used by parents with fewer years of formal education (70 % *v*. 61 %; *P* = 0·04), and those parents also more frequently used media as a source of information compared to parents with more years of formal education (33 % *v*. 22 %; *P* = 0·007). Primiparous *v*. multiparous parents more frequently used their parental network (75 % *v*. 55 %; *P* < 0·001) and the internet (81 % *v*. 66 %; *P* < 0·001) (Table 4).

#### Infant and young child feeding perceptions and information-seeking practices of parents of children with medical condition *v.* parents of healthy children

Parents of CMC (*n* 175) perceived that CoF did not go as well as it did for parents of healthy children (86 % *v*. 92 %; *P* = 0·01) and was more frequently a source of concern (56 % *v*. 31 %; *P* < 0·001). They also reported they were less informed about IYCF (81 % *v*. 88 %; *P* = 0·02), the available information was more contradictory (51 % *v*. 32 %; *P* < 0·001), and it gave more of a perception that they were not implementing IYCF correctly (45 % *v*. 30 %; *P* < 0·001) (online supplementary material, Supplemental Table 1, Supplemental Material 3).

The mean age of the child when CMC parents began to look for information on IYCF was 4·9 ± 4·1 months (median = 4 months; IQR 3–6 months). Eighty percentage of CMC parents reported that they received advice from HCP during follow-up consultations, that is, more frequently than parents of healthy children. Parents of CMC generally looked for the same content as parents of healthy children, except for examples of recipes/menus, for which they searched less frequently (53 % *v*. 63 %; *P* = 0·01, online supplementary material, Supplemental Table 2, Supplemental Material 3).

Parents of CMC more frequently used the internet (80 % *v*. 72 %; *P* = 0·03) and media sources (37 % *v*. 24 %; *P* < 0·001) to inform themselves about IYCF compared with parents of healthy children. CMC parents were slightly more influenced by media and childcare professionals (online supplementary material, Supplemental Tables 3 and 4, Supplemental Material 3).

## Discussion

The aim of this study was to explore the information-seeking practices, needs and determinants of French parents with regard to IYCF (including CoF) using a nationally representative sample. The parents were aware of the importance of IYCF for the health and growth of their children. First-time parents were generally more insecure when facing CoF; they found more contradictions when looking for guidance, and they were less satisfied with the information. As previously shown in other studies, parents accessed information about IYCF from a wide variety of sources^(30,31)^, confirming the complexity of the information environment^(15)^. The most used and trustworthy source was HCP, followed by the internet, which was the least influential source. Those sources had a very different reported influence on parental feeding behaviour, and the relative influence could differ according to the studied characteristics of the parent (financial situation, education and parity) or the child (age, prematurity). Lastly, the parents often looked for practical tips regarding IYCF, such as examples of recipes and menus.

The latest advances in new communication and information technologies have revolutionised the way health information is gathered and disseminated^(32)^. Currently, a massive quantity of information is reachable via different sources, including the internet, where social media use has expanded among mothers looking for advice on childhood health^(33)^. However, the quantity and accessibility of this information are not necessarily related to the level of understanding of the messages^(34,35)^. Our results show that the parents of 2020 felt well-informed and satisfied with the available IYCF information; they also found this information easy to understand and put into practice. This finding was especially true for multiparous parents and those with older children. Those parents could also feel more confident because of their previous experience, as reported in different studies^(36,37)^. Nevertheless, for one-third of the parents in our study, the available information could appear contradictory and give the perception they were not implementing IYCF correctly. This observation highlights the room for improvement regarding the formulation of public health messages regarding IYCF.

Our results suggest that parents recognise HCP as their most influential source of advice and the option to which they refer most often to obtain information on IYCF. This perspective was especially true for parents of younger children, those with a better financial situation or with a higher education level. The vast use of this professional source could be related to the fact that in France, several free and mandatory consultations with HCP take place when the child is between 0 and 16 years old, with the majority of the visits occurring within the first 3 years of life^(38)^. French HCP are in a dominant position to disseminate IYCF information. Previous studies investigating the role played by different sources in influencing mothers’ feeding decisions across countries have shown that cultural- and country-related factors impact how mothers use information^(22,39)^. For example, Gage *et al.* found that lower proportions of mothers in England and Finland are influenced by doctors in making infant feeding decisions compared with mothers in Germany, Hungary and Spain, reflecting the different roles played by HCP depending on the public health policies of each country^(22)^. Our study shows that parents of CMC resort as frequently as parents of healthy children to HCP. The use of the internet to establish contact with others in similar situations to exchange opinions has been reported in the literature and it could apply to our study^(40)^. This observation suggests a specific role for forums in a public health communication strategy for parents with special needs. A deeper investigation of IYCF information in terms of the searching practices of CMC parents and accounting for specific feeding-related illnesses might be of help in defining to what extent a generic communication strategy can be suited to their needs.

The internet is a widely used source of information according to our results; however, its degree of influence is the lowest of all the examined sources. The internet was used more by and had more influences on parents of younger children, and it was used more by primiparous than multiparous parents. First-time mothers are the most active in looking up information on the internet, according to a Swedish literature review^(40)^. The reasons why new parents (or parents) seek information on the internet could vary. Performing research on the internet is easy and allows parents to gather answers to their questions quickly regardless of where they are or the time at which the information is needed^(41,42)^. Using the internet, it is easier to find the most updated information, and parents can find both expert advice and peer support (via blogs, forums)^(40)^. Looking for information on the internet could also help parents integrate or complete the information given to them by HCP during a quick consultation^(43)^; moreover, searches can be performed anonymously with regard to some topics that can be embarrassing to discuss with the doctor^(40)^.

The internet has the potential of being a largely accessible source of information, but being able to perform meaningful searches has been shown to require the development of specific competencies in terms of eHealth literacy^(44)^. One of the reasons is that the credibility and truthfulness of the given information require constant verification. Furthermore, recent studies have explored the problem of the digital divide, showing that socio-economic disparities are present in the access to and use of health information, especially with regard to internet use^(45)^. However, the present study does not reveal differences in the use or influence of the internet according to financial situation or education level. In France, Banti *et al.* found that the information on websites dedicated to CoF could be contradictory and not always consistent with that of the French Society of Pediatrics, showing the need for a recognised official website^(46)^.

According to our results, the financial situation of the parents was not a determinant of the source used to gather information on IYCF. Nevertheless, there were some differences in regard to searched topics based on financial situation and level of education. Parents with more vulnerable financial situations looked more frequently for information on menus/recipes and less often for information on promoting the development of healthy eating habits or the appropriate age for the introduction of foods. They were less influenced by HCP. Parents with more years of formal education better recognised the importance of IYCF for the health of their child; they looked more often for information regarding feeding strategies and portion sizes. This approach highlights the need for public health communication content that is easily accessible and understandable for parents with lower socio-economic positions to avoid reinforcing social disparities in health.

Our study confirms that first-time parents and parents with fewer years of formal education often use their personal network as a source of information^(11,47)^. Surprisingly, parents with more years of formal education are more influenced by this informal source than parents with fewer years of formal education despite a lower level of use. This observation is in contrast with a study conducted in the UK on mothers with more years of formal education showing that advice on CoF was commonly received from friends and family but was often perceived as negative and outdated^(48)^. The high influence of personal network advice could be related to the fact that parents become overwhelmed and are left confused about the large amount of conflicting information; thus, they end up adopting valued familial or culturally established practices or choosing the advice that best suits them^(49)^. Recognising the primary influencers of parents and better understanding familial transmission regarding IYCF information is of interest but beyond the scope of the current study.

Paper-based support sources were used by 44 % of the parents in our study, and not surprisingly, parents with more years of formal education reported that they were more influenced by this source^(35)^, whereas parents with fewer years of formal education reported using media more. For parents of 2020 in France, paper documents still have a role to play in a digital world. The parents in this study gave credit for the information obtained from childcare professionals, especially when the child was born preterm, but they made little use of this source.

Our study confirms that parents, especially those in difficult financial situations and with lower levels of education, need practical advice regarding IYCF^(23)^. Practical needs are identified not only as ideas for menus/recipes but also as feeding strategies (e.g. addressing ‘little’ or ‘big’ appetites). This last topic is researched more by parents with more years of formal education and parents of older children (12–35 months old), which could be related to the fact that as the child grows, neophobia develops, and parents begin to face food refusal^(50)^. Those parents may also have a higher level of concern regarding their children’s future weight status and may want to control their child’s appetite^(51)^.

No differences were reported in terms of content research for parents of premature *v*. full-term children. This finding is surprising since parents of preterm-born children could base their feeding decisions on corrected age, not chronological age. For that reason, it might be natural to think that questions about the age of introduction of the first foods or different food groups may easily arise in parents, but this is not what our findings show. Developmental considerations may lead to think that preterm-born children could be ready to eat solids later than their norm-term peers, even if there is a lack of evidence regarding the optimal age or the best first foods to introduce into their diet^(52)^. However, a recently published systematic review showed that premature infants are generally introduced to solids earlier than norm-term infants, often because breast-feeding or milk feeding can be challenging with them^(53)^. Whether premature babies are actually ready at the same postnatal age as norm-term babies remains a topic that requires further clarification to promote the relevant public health messages.

Undifferentiated access to many sources and the individual ability to comprehend information can impact the completion of parents’ information needs. In the literature, the notion of parental health literacy is widely explored, showing that the individual capacity to obtain, process and understand basic health information might influence the information-seeking strategies of parents and subsequently their health knowledge and behaviours^(54,55)^. This phenomenon can accentuate health inequalities, which are also related to socio-economic disparities; lower parental health literacy is associated with specific obesogenic infant care behaviours^(56,57)^. However, providing information with the only aim of increasing knowledge has been shown to be insufficient to spur the adoption of health-related behavioural changes^(58)^. Because the successful implementation of public health practices depends largely on behaviour changes, interactive communication approaches and the simultaneous use of different theories and strategies for behaviour change are necessary to encourage people to follow recommendations. The theoretical framework designed by Michie and colleagues (Behaviour Change Wheel) shows that effective interventions should cover multiple intervention functions and policies and should address several drivers within capability, motivation or opportunity^(59)^. Providing information is related to capability building and is one of the most frequently used behavioural change techniques for infant feeding interventions^(60,61)^, but it is just the start of a process of facilitating the adoption of healthy feeding behaviour. Further research may complement the present findings to address other components of behavioural change.

Concern should be raised for public health communicators regarding the need to find the best solution to having the entire population on the same level when looking for health information, especially on the internet, thus ensuring that parents with a lower health literacy level can easily retrieve information. Attention should be paid to indexing official websites in search engines so that evidence-based information can reach everyone equally. Another takeaway from our study is that public health stakeholders could consider making better use of childcare professionals as a means of transmitting information based on the influence that these figures have on parents and the regular contacts they have during the very first months of the child’s life. The provision of evidence-based information, as translated into easy messages that could be understood by the largest part of the population of interest and spread via easily accessible sources, should serve as the foundation for a more structured education strategy that is ultimately aimed at the adoption of healthy feeding-related behaviours starting from infancy.

Our findings add to the knowledge about the multiple sources of IYCF information available to parents and their influence. However, the study strengths and limitations must be considered. The limitations of the study lie in its nature, because it did not make a deeper exploration of possible motivations or the understanding of the health messages. Additionally, the use of closed-ended questions facilitates data analysis but limits the possibility of exploring contradictions. Complementing the study with a qualitative section could have allowed us to deepen some aspects of this work. The primary strength of the study lies in its use of a large, nationally representative French sample. This makes the generalisation of the results to the French population of young parents possible, even if using the quota sampling method and voluntary online surveys has sometimes been noted as a source of potential bias due to the limited inclusion of populations in precarious situations. The methodology deployed here can also be used in other countries, provided that some cultural adaptations are made to the questionnaire. Moreover, we explored information-seeking strategies during the IYCF period and through the completion of the third year, which is not frequent in the literature. In fact, to the best of our knowledge, this topic is more often investigated during the antenatal, pregnancy or milk-feeding periods than during IYCF and CoF.

## Conclusions

Parents receive IYCF information from multiple sources, which can lead to confusion when deciding which advice to follow. We highlighted differences in search strategies according to parity or child age, but surprisingly, few differences among parents of premature *v*. full-term children were found. This study contributes to the evidence available for public health stakeholders when updating and providing resources for parents regarding IYCF. Dissemination via HCP and childcare professionals is preferable due to their influence on parental behaviour. Attention should be paid to the clarity of the explanation on the content, making sure to adopt a tone that is less injunctive and that the content will be adapted to parents with different socio-economic statuses to avoid accentuating health literacy inequalities. In developing communication strategies for IYCF guidelines, both paper-based and digital tools should be considered, including the creation of a recognised official website that is well-indexed in search engines or a digital tool such as an application for smartphones. These tools should help confront topics related to practical needs. Our findings will ultimately help to build a new French public health communication strategy regarding IYCF (0–3 years old) by taking advantage of the IYCF sources that most influence parents.

## Supplementary material

For supplementary material accompanying this paper visit https://doi.org/10.1017/S1368980021003086. click here to view supplementary material
