# Supplementary material for: Information seeking of French parents regarding infant and young child feeding: practices, needs and determinants
Source: Public Health Nutr. 2021 Jul 29;25(4):879–92. doi: 10.1017/S1368980021003086 (PMC9991613; doi:10.1017/S1368980021003086)
Supplement: Supplementary file 1 [file S1368980021003086sup.zip › S1368980021003086sup001.pdf]

Supplement Material 1. Studied characteristics, questions and their modalities.

Characteristics	Modalities
**Youngest child's characteristics**	
Sex	Female; male
Age	<12 months; 12-23 months; 24-35 months; ≥36 months
Born pre term	Yes (birth at 36 weeks or less); no (birth at 37 weeks or more)
Have or had a medical condition affecting the diet	Yes (for example gastroesophageal reflux treated by medication, allergy to cow's milk protein, nasogastric intubation or digestive tract congenital defects); no
Eating food other than milk	Yes (CoF started); no (CoF had not started yet)
**Parental demographic and socioeconomic characteristics**	
Sex	Female; male
Age	<35 years old; ≥35 years old
Education level	<A level (did not attend university); ≥A level (attended university).
Socio-professional category of the interviewed parent	High (Self-employed business owners, Executive, Intermediate); Low (Manual worker, Office employee); no occupation/retired.
Parity	Primiparous (1 child 0-3 years old) ; Multiparous (>1 child, the youngest child is 0-3 years old)
Mother tongue	French; other
**Household characteristics**	
Self-perception of financial situation	Good (I am comfortable, I am OK?); difficult (ok but I need to be careful, difficult to get the end of the month, I cannot make it without debts); no answer
Living area	Rural (<20000 inhabitants); urban (>20000 inhabitants)
Socio-professional category of the household reference parent †	High (Self-employed business owners, Executive, Intermediate); low (Manual worker, Office employee); no occupation/retired.
**Perceptions of IYCF (including CoF) (**the following statements were presented and answers were recorded on a 4-point Likert scale grouped in agree and disagree for the analysis)<br>(the definition of CoF was given to parents when they had to answer a question related to that)	
1) CoF for my youngest child is going or went well	Strongly agree; Tend to agree (=Agree); Tend to disagree; Strongly disagree (=disagree)
2) It is easy to find information on IYCF (including CoF)	Strongly agree; Tend to agree (=Agree); Tend to disagree; Strongly disagree (=disagree)
3) CoF is easier for my last child than for the first one(s) (only	Strongly agree; Tend to agree (=Agree); Tend to disagree; Strongly disagree (=disagree)

	multiparous)	
4)	CoF for my last child is or it has been source of concern	Strongly agree; Tend to agree (=Agree); Tend to disagree; Strongly disagree (=disagree)
5)	IYCF is important for the present and future health of my child and for his/her growth	Strongly agree; Tend to agree (=Agree); Tend to disagree; Strongly disagree (=disagree)
6)	IYCF is important for the establishment of good eating habits	Strongly agree; Tend to agree (=Agree); Tend to disagree; Strongly disagree (=disagree)
**Perceptions of IYCF information (including CoF)** (the following statements were presented and answers were recorded on a 4-point Likert scale grouped in agree and disagree for the analysis)		
7)	I feel well informed about IYCF	Strongly agree; Tend to agree (=Agree); Tend to disagree; Strongly disagree (=disagree)
8)	I am satisfied with the available information on IYCF (including CoF)	Strongly agree; Tend to agree (=Agree); Tend to disagree; Strongly disagree (=disagree)
9)	The available information on IYCF (including CoF) answers to my questions	Strongly agree; Tend to agree (=Agree); Tend to disagree; Strongly disagree (=disagree)
10)	The available information on IYCF (including CoF) is clear, easy to understand	Strongly agree; Tend to agree (=Agree); Tend to disagree; Strongly disagree (=disagree)
11)	The available information on IYCF (including CoF) is easy to put into practice	Strongly agree; Tend to agree (=Agree); Tend to disagree; Strongly disagree (=disagree)
12)	The available information on IYCF (including CoF) is contradictory	Strongly agree; Tend to agree (=Agree); Tend to disagree; Strongly disagree (=disagree)
13)	The available information on IYCF (including CoF) is giving me the perception of not implementing correctly IYCF	Strongly agree; Tend to agree (=Agree); Tend to disagree; Strongly disagree (=disagree)
**Information sources used by parents** (multiple choice answer: if parents choose the main source on the left column they had the possibility to select between the options on the right column) **and how strongly the sources influenced parents in making decisions in feeding their youngest child** (linear scale from 1 to 10: 1 means the source did not influence decisions at all and 10 means it influenced decisions very strongly).		
Health care professionals		Pediatricians; General practitioners; Midwives; Pediatric nurses; Dieticians
Internet, social networks or applications for smartphones		Website specialized in early childhood; Blogs or forums for parents; Website specialized in health ; Websites of baby food manufacturer/industries; Health authority website; Social networks; Applications for smartphones; Health insurance websites

Parents' network	Own parents; Friends; Own grandparents; Other family's members
Paper tools	Books; Printed media, newspapers, magazines
Early childhood professionals	Social service for mothers ("Personnel de PMI"); Licensed childminder
Media	Television, radio
**Type of information sought by parents** (multiple choice answer: if parents choose the main topic on the left column they had the possibility to select between the options on the right column)	
Menus, recipes	Example of menus<br>Example of recipes
Age and modalities of introduction of	Different food groups<br>First food pieces<br>First foods other than milk
Feeding strategies	How to present food in case of food refusal<br>How to deal with little appetite of the child<br>How to deal with big appetite of the child
Portions sizes	Portions sizes of food<br>Adapt milk quantities when introducing other foods
How to feed a child to promote the development of healthy eating habits	
How to interpret the child hunger's and satiation's cues	

IYCF, infant and young child feeding. CoF, complementary feeding. HCPs, health care professionals.
† i.e., the parent with the highest income.
