# Supplementary material for: Information seeking of French parents regarding infant and young child feeding: practices, needs and determinants
Source: Public Health Nutr. 2021 Jul 29;25(4):879–92. doi: 10.1017/S1368980021003086 (PMC9991613; doi:10.1017/S1368980021003086)
Supplement: Supplementary file 1 [file S1368980021003086sup.zip › S1368980021003086sup002.pdf]

Supplement Material 3. Infant and young child feeding (IYCF) perceptions and information seeking practices of French parents of children with medical condition that could affect his/her diet vs. parents of healthy children: tables with results.

Supplement Table 1. Differences in perceptions (frequencies and percentages of parents who answered positively for each item) of IYCF (including CoF) and of IYCF information between parents HC (n=826) and parents of CMC (n=175)

	N (%)		
	**Parents of HC**	**Parents of CMC**	**p-value***
**All**	826 (83)	175 (17)	
**Perceptions on IYCF (including CoF)**			
CoF for my youngest child is going well it or went well	762 (92)	151 (86)	**0.01**
It is easy to find information on IYCF (including CoF)	727 (88)	146 (83)	0.10
CoF is easier for my last child than for the first one (only multiparous, n=613)	269 (53)	65 (61)	0.15
CoF for my last child is or it has been source of concern	252 (31)	98 (56)	**< 0.001**
IYCF is important for the present and future health of my child and for his growth	815 (99)	173 (99)	0.84
IYCF is important for the establishment of good eating habits	819 (99)	170 (97)	**0.03**
**Perceptions on IYCF information**			
I feel well informed about IYCF	726 (88)	142 (81)	**0.02**
I am satisfied with the available information on IYCF (including CoF)	714 (86)	151 (86)	0.96
The available information on IYCF (including CoF) answers to my questions	723 (86)	150 (86)	0.51
The available information on IYCF (including CoF) is clear, easy to understand	744 (90)	151 (86)	0.14
The available information on IYCF (including CoF) is easy to put into practice	710 (86)	149 (85)	0.78
The available information on IYCF (including CoF) is contradictory	260 (32)	89 (51)	**< 0.001**
The available information on IYCF (including CoF) is giving me the perception of not implementing correctly IYCF	251 (30)	78 (45)	**< 0.001**

IYCF: Infant and young child feeding; CoF: complementary feeding. HC: healthy children; CMC: children with medical condition that could affect the diet.

This table shows frequencies and percentages of parents, that answered positively (strongly agree and tend to agree) to the statements in the left column.

* based on $\chi 2$ tests, comparing perceptions and feelings of parents of HC and parents of CMC. Significant p-values are in boldface.

Supplement Table 2. Differences in type of content searches between parents of HC (n=826) and parents of CMC (n=175) (frequencies and percentages of parents, that answered positively)

	Parents of HC	Parents of CMC	p-value*
**All**	**826**	**175**	
Subtotal Menus, recipes	523 (63)	93 (53)	**0.01**
Subtotal Age and modalities of introduction	442 (53)	97 (55)	0.64
Subtotal Feeding strategies	435 (53)	97 (55)	0.51
Subtotal Portion sizes	452 (55)	100 (57)	0.56
How to feed a child to promote the development of healthy eating habits	258 (31)	47 (27)	0.25
How to interpret the child hunger's and satiety's cues	207 (25)	42 (24)	0.77

HC: healthy children. CMC: children with medical conditions.
* based on $\chi 2$ tests, comparing parents of HC and parents of CMC. Significant p-values are in boldface.

Supplement Table 3. Differences in the use of IYCF information sources between parents of HC (n=826) and parents of CMC (n=175) (frequencies and percentages of parents, that answered positively = declared using that source)

	Parents of HC	Parents of CMC	p-value*
**All**	**826**	**175**	
Health care professionals	669 (81)	143 (82)	0.82
Internet	594 (72)	140 (80)	**0.03**
Parent's network	517 (63)	106 (61)	0.62
Paper	360 (44)	78 (45)	0.81
Childcare professionals	251 (30)	63 (36)	0.15
Medias	197 (24)	65 (37)	**< 0.001**

IYCF: infant and young child feeding. HC: healthy children. CMC: children with medical conditions.
* based on $\chi 2$ tests, comparing parents of HC and parents of CMC. Significant p-values are in boldface.

Supplement Table 4. Differences in the influence of IYCF information sources between parents of HC (n=826) and parents of CMC (n=175). Means (± SDs) of the influence of the sources that parents declared to use (parents rated sources on a scale from 1 to 10 and this variable was considered as continuous).

	Parents of HC	Parents of CMC	p-value*
Health care professionals	7.7 ± 1.7	7.5 ± 1.9	0.44
Internet	5.6 ± 2.1	6.0 ± 2.3	0.07
Parent's network	6.9 ± 1.8	7.2 ± 1.8	0.10
Paper	6.2 ± 1.8	6.6 ± 2.1	0.06
Childcare professionals	7.3 ± 1.8	7.9 ± 1.5	**0.04**
Medias	5.8 ± 2.0	6.6 ± 2.0	**0.01**

IYCF: infant and young child feeding. HC: healthy children. CMC: children with medical conditions.
* based on T-tests to compare means of parents of HC and parents of CMC. Significant p-values are in boldface.
