# Supplementary material for: Information seeking of French parents regarding infant and young child feeding: practices, needs and determinants
Source: Public Health Nutr. 2021 Jul 29;25(4):879–92. doi: 10.1017/S1368980021003086 (PMC9991613; doi:10.1017/S1368980021003086)
Supplement: Supplementary file 1 [file S1368980021003086sup.zip › S1368980021003086sup003.pdf]

Supplement Material 2. Original questionnaire.

Enquête sur les parents de jeunes enfants et la diversification alimentaire
Questionnaire

Rappel de la méthodologie

Enquête par internet auprès d'un échantillon national représentatif de 1000 parents d'enfant de moins de 4 ans.
La représentativité de l'échantillon sera assurée par la méthode des quotas appliquée aux variables suivantes : âge du parent, CSP de la personne référente du ménage, région, catégorie d'agglomération, parent primipare ou non.

Renseignements signalétiques/quotas

Z9. Combien d'enfants avez-vous ?
Si vous n'en avez pas, indiquer 0
/__/__/ enfant(s)

Z10. Précisément quel est le mois et l'année de naissance ainsi que le sexe de chacun de vos enfants ?
a) 1er enfant : Mois : /__/__/ Année /__/__/__/__/ sexe : 1. Homme 2. Femme
b) 2ème enfant : Mois : /__/__/ Année /__/__/__/__/ sexe : 1. Homme 2. Femme
c) 3ème enfant : Mois : /__/__/ Année /__/__/__/__/ sexe : 1. Homme 2. Femme
d) …

Z1. Êtes-vous :
1. Un homme
2. Une femme

Z2. Quel est votre âge ?
/__/__/ ans

Z0a. Merci d'indiquer le code postal de votre commune
/__/__/__/__/__/

Z0b. Dans quelle commune habitez-vous ?

Z3. En ce moment, quelle est votre activité principale ?
1. Salarié du secteur privé
2. Salarié d'une entreprise publique ou nationale
3. Salarié du secteur public
4. A votre compte
5. A la recherche d'un premier emploi
6. A la recherche d'un emploi (vous avez déjà travaillé)
7. A la retraite
8. Au foyer

9. Elève ou étudiant
10. Dans une autre situation

Z3a. Actuellement, travaillez-vous à…

1. temps complet
2. temps partiel

Z3b. Quelle est votre profession, votre catégorie socio-professionnelle ?

Z3b. Avant d'être au chômage, quelle était votre dernière profession, votre dernière catégorie socio-professionnelle ?

1. Agriculteur exploitant
2. Artisan petit commerçant
3. Chef d'entreprise de plus de 10 salariés
4. Profession libérale (SAUF paramédical)
5. Professeur / profession scientifique
6. Cadre et autre profession intellectuelle supérieure
7. Contremaître, agent de maîtrise, profession paramédicale, technicien
8. Instituteur
9. Employé
10. Personnel de service
11. Ouvrier / Ouvrier agricole
12. Retraité
13. Élève / étudiant
14. Autre inactif

Z4. Êtes-vous la personne de référence du ménage ?

La personne de référence est la personne en activité au sein de votre foyer qui apporte le plus de revenus

1. Oui
2. Non

Z8. En ce moment, quelle est l'activité principale de la personne de référence du ménage ?

La personne de référence est la personne en activité au sein de votre foyer qui apporte le plus de revenus

1. Salarié du secteur privé
2. Salarié d'une entreprise publique ou nationale
3. Salarié du secteur public
4. A son compte
5. A la recherche d'un premier emploi
6. A la recherche d'un emploi (a déjà travaillé)
7. A la retraite
8. Au foyer
9. Élève ou étudiant
10. Dans une autre situation

Z8b. Quelle est la profession, la catégorie socio-professionnelle de la personne de référence du ménage ?

Z8b. Avant d'être au chômage, quelle était la dernière profession, la dernière catégorie socio-professionnelle de la personne de référence du ménage ?
La personne de référence est la personne en activité au sein de votre foyer qui apporte le plus de revenus

1. Agriculteur exploitant
2. Artisan petit commerçant
3. Chef d'entreprise de plus de 10 salariés
4. Profession libérale (SAUF paramédical)
5. Professeur / profession scientifique
6. Cadre et autre profession intellectuelle supérieure
7. Contremaître, agent de maîtrise, profession paramédicale, technicien
8. Instituteur
9. Employé
10. Personnel de service
11. Ouvrier / Ouvrier agricole
12. Retraité
13. Élève / étudiant
14. Autre inactif

Z6. De combien de personnes se compose votre foyer, vous y compris ?

1. 1 personne
2. 2 personnes
3. 3 personnes
4. 4 personnes
5. 5 personnes
6. 6 personnes
7. 7 personnes
8. 8 personnes
9. 9 personnes ou plus

Vécu et comportements avec l'enfant le plus jeune

Cette enquête concerne principalement VOTRE ENFANT LE PLUS JEUNE. Merci de bien lire attentivement toutes les questions et d'en tenir compte lorsque vous répondez.

Q1. Votre plus jeune enfant est né…

1. À terme (accouchement à 37 semaines ou plus)
2. Prématuré (accouchement à 36 semaines ou moins)

Q2. Votre plus jeune enfant a-t-il été allaité au sein (tire-lait inclus) ?

1. Oui
2. Non

Q3. Combien de temps a-t-il été allaité au sein (tire-lait inclus) ?

1. /__/ jours
2. /__/ semaines
3. /__/ mois
4. Je ne sais pas

Q4. Est-ce que votre plus jeune enfant a commencé à manger des aliments autres que le lait ?

1. Oui
2. Non

Q5. Actuellement, à quelle fréquence donnez-vous à votre plus jeune enfant…

a) des plats ou petits pots pour bébé achetés dans le commerce ?
b) des plats ou des petits pots maison que vous avez cuisinés ?

1. Systématiquement
2. Souvent
3. De temps en temps
4. Jamais

Q6. Depuis la naissance de votre plus jeune enfant, lors de rendez-vous médicaux habituels avec des professionnels de santé (pédiatre, médecin, infirmier…), vous ont-ils donné spontanément des conseils sur l'alimentation de votre enfant (aliments autre que le lait) dont la diversification alimentaire ?

La diversification alimentaire est la période pendant laquelle des aliments autres que le lait sont progressivement introduits dans l'alimentation d'un enfant

1. Régulièrement, tout au long du suivi de mon enfant
2. Plusieurs fois pendant la période de diversification alimentaire
3. Uniquement au début de la diversification alimentaire
4. Jamais

Q7. Depuis la naissance de votre plus jeune enfant, lors de rendez-vous médicaux habituels avec des professionnels de santé (pédiatre, médecin, infirmier…), leur avez-vous vous-même posé des questions ou demandé des conseils sur l'alimentation de votre enfant (aliments autre que le lait) dont la diversification alimentaire ?

La diversification alimentaire est la période pendant laquelle des aliments autres que le lait sont progressivement introduits dans l'alimentation d'un enfant

1. Régulièrement, tout au long du suivi de mon enfant
2. Plusieurs fois pendant la période de diversification alimentaire
3. Uniquement au début de la diversification alimentaire
4. Jamais

Q8. Votre plus jeune enfant a-t-il ou a-t-il eu un problème de santé pouvant affecter fortement son alimentation, comme par exemple : reflux gastro œsophagien pris en charge médicalement, allergie aux protéines de lait de vache, intubation nasogastrique, anomalies congénitales du tube digestif ?

1. Oui

2. Non

Sentiment d'information vis-à-vis de l'alimentation des enfants de 0 à 3 ans

Q9. Aujourd'hui, vous sentez-vous bien informé sur ce qu'un enfant de 0 à 3 ans peut manger en dehors du lait ?

1. Oui, tout à fait
2. Oui, plutôt
3. Non, plutôt pas
4. Non, pas du tout

Q10. Selon vous, l'alimentation de 0 à 3 ans (autre que le lait) est-elle importante pour la santé actuelle et future des enfants et leur croissance ?

1. Très importante
2. Plutôt importante
3. Plutôt pas importante
4. Pas du tout importante

Q11. Selon vous, l'alimentation de 0 à 3 ans (autre que le lait) est-elle importante pour que les enfants aient ensuite de bonnes habitudes alimentaires ?

1. Très importante
2. Plutôt importante
3. Plutôt pas importante
4. Pas du tout importante

Q12. Globalement, êtes-vous satisfait des informations sur l'alimentation de 0 à 3 ans (autre que le lait) dont la diversification alimentaire, que vous avez à votre disposition ?

La diversification alimentaire est la période pendant laquelle des aliments autres que le lait sont progressivement introduits dans l'alimentation d'un enfant

1. Très satisfait
2. Plutôt satisfait
3. Plutôt pas satisfait
4. Pas du tout satisfait

Q13. Et globalement concernant l'alimentation de 0 à 3 ans (autre que le lait) dont la diversification alimentaire, diriez-vous que les informations et conseils que vous avez à votre disposition sur ce sujet…

La diversification alimentaire est la période pendant laquelle des aliments autres que le lait sont progressivement introduits dans l'alimentation d'un enfant

a) Répondent à vos questions
b) Sont clairs, faciles à comprendre
c) Sont faciles à mettre en pratique pour votre enfant
d) Se contredisent, ne sont pas cohérents entre eux
e) Sont culpabilisants pour les parents

1. Oui, tout à fait
2. Oui, plutôt
3. Non, plutôt pas

4. Non, pas du tout

Q14. Êtes-vous d'accord ou non avec les affirmations suivantes concernant la diversification alimentaire ?

La diversification alimentaire est la période pendant laquelle des aliments autres que le lait sont progressivement introduits dans l'alimentation d'un enfant

a) Il est facile de trouver des informations concernant la diversification alimentaire
b) La diversification alimentaire de mon plus jeune enfant est ou a été source d'inquiétude
c) La diversification alimentaire pour mon plus jeune enfant se passe bien ou s'est bien passée
d) La diversification alimentaire est plus facile pour mon dernier enfant que pour le premier

1. Tout à fait d'accord
2. Plutôt d'accord
3. Plutôt pas d'accord
4. Pas du tout d'accord

Sources d'informations et recherche

Q15. Au cours des 12 derniers mois, par quels moyens avez-vous eu des informations sur l'alimentation de votre plus jeune enfant (alimentation autre que le lait) ?

Plusieurs réponses possibles

Média

1. Télévision
2. Radio

Support ou document papier

3. Presse écrite, journaux, magazines
4. Livres

Internet, réseaux sociaux ou applications smartphone

5. Blog ou forum de parents
6. Application pour smartphone
7. Site internet d'industriel ou fabricant d'aliments pour bébé (bledina, nestlé, babybio, hipp, goodgout…)
8. Site internet spécialisé dans la petite enfance (parents.fr, magicmaman.com…)
9. Site internet dédié à la santé (doctissimo.fr, santémagazine.fr, topsanté.com, passeportsanté.net…)
10. Site internet d'une autorité de santé (ameli.fr, mangerbouger.fr, santépubliquefrance.fr, santé.gouv.fr, santé.fr…)
11. Site internet d'une mutuelle ou d'une assurance (lamutuellegenerale.fr, mutualite.fr, axaprevention.fr, harmonie-mutuelle.fr, interiale.fr…)
12. Réseaux sociaux (facebook, instagram, twitter, youtube, whatsapp…)
13. Autre site internet

Entourage

14. Mes amis
15. Mes parents
16. Mes grands-parents
17. Un autre membre de la famille

Professionnel de santé

18. Médecin généraliste
19. Pédiatre
20. Infirmier(ère), puériculteur(trice)
21. Diététicien(ne)
22. Sage-femme

Professionnel de la petite enfance

23. Personnel de PMI ou d'établissement d'accueil de jeunes enfants (crèche, accueil collectif…)
24. Assistante maternelle

25. Autre moyen, précisez : ____________________

Q16. Sur quel(s) réseau(x) social(ux) vous êtes-vous informé concernant l'alimentation de votre plus jeune enfant (aliments autres que le lait) ?

Plusieurs réponses possibles

1. Facebook
2. Instagram
3. Twitter
4. Youtube
5. Whatsapp
6. Autre

Q17. Vous arrive-t-il de partager sur les réseaux sociaux, sur des blogs ou des forums, des informations sur l'alimentation de l'enfant de 0 à 3 ans que vous trouviez utiles ?

1. Très souvent
2. Souvent
3. De temps en temps
4. Jamais

Q18. Pour chacune de vos sources d'information, indiquez sur une échelle de 1 à 10 dans quelle mesure elles ont influencé vos décisions sur la façon de nourrir votre plus jeune enfant au cours des 12 derniers mois.

1 signifie qu'elle n'a pas du tout influencé vos décisions et 10 signifie qu'elle a très fortement influencé vos décisions. Les notes intermédiaires permettent de nuancer votre jugement.

a) La télévision ou la radio
b) Les supports ou documents papier comme la presse écrite, les journaux, les magazines ou les livres
c) Internet, réseaux sociaux ou applications smartphone
d) L'entourage
e) Les professionnels de santé
f) Les professionnels de la petite enfance

N'a pas du tout influencé mes décisions											A très fortement influencé mes décisions
	1	2	3	4	5	6	7	8	9	10	

Q19. Vous est-il arrivé de chercher des informations concernant l'alimentation des enfants de 0 à 3 ans (autre que le lait) pour votre enfant ?

1. Oui
2. Non

Q20. Quel âge avait votre plus jeune enfant lorsque vous avez commencé à chercher des informations sur l'alimentation autre que le lait ?

S'il avait moins d'1 mois, indiquer 0

/__/__/ mois

Confiance dans les sources d'informations

Q21. Pour avoir une information fiable sur l'alimentation des enfants de 0 à 3 ans (autre que le lait), faites-vous personnellement confiance ou non…

a) Aux médias comme la télévision, la radio ou la presse écrite
b) Aux livres dédiés à la petite enfance
c) À Internet
d) Aux réseaux sociaux, blogs ou forums de parents
e) Aux autorités de santé ou pouvoirs publics
f) Aux industriels ou fabricants de produits alimentaires pour jeunes enfants
g) Aux professionnels de santé (médecin, pédiatre, infirmière, sage-femme…)
h) Aux professionnels de la petite enfance (personnel de PMI ou d'établissement d'accueil des jeunes enfants, assistante maternelle…)

1. Tout à fait confiance
2. Plutôt confiance
3. Plutôt pas confiance
4. Pas du tout confiance

Attentes en matière de type et de format d'informations

Q22. Quels types d'informations sur l'alimentation des enfants de 0 à 3 ans (autre que le lait) et la diversification alimentaire vous seraient particulièrement utiles pour votre enfant le plus jeune ?

5 réponses maximum

1. Âge d'introduction des premiers aliments autres que le lait
2. Âge d'introduction des différents groupes d'aliments
3. Âge et modalités d'introduction des premiers morceaux d'aliments
4. Comment donner ou présenter les aliments en cas de refus
5. Quantités et tailles des portions à proposer à l'enfant
6. Stratégies alimentaires en cas de « petit appétit » de l'enfant
7. Stratégies alimentaires en cas de « gros appétit » de l'enfant
8. Comment interpréter les signaux de l'enfant lorsqu'il mange ou refuse de manger
9. Comment nourrir un enfant pour favoriser le développement d'habitudes alimentaires saines
10. Exemples de recettes
11. Exemples de menus

12. Adaptation de la quantité de lait à l'introduction des autres aliments
13. Autres informations, précisez :_________

Q23. Pour vous, quels types de supports sont les plus adaptés pour avoir des informations sur l'alimentation des enfants de 0 à 3 ans (autre que le lait) dont la diversification alimentaire ?

3 réponses maximum

1. Une brochure papier d'1 page
2. Un livret papier de plusieurs pages
3. Un site internet
4. Une application pour smartphone
5. Un espace interactif sur internet avec la possibilité de poser des questions (chat, blog, forum, post de témoignage…)
6. Autre, précisez :_________

**Autres renseignements signalétiques**

Q24. Êtes-vous né en France ?

1. Oui
2. Non

Q25. Depuis combien de temps habitez-vous en France ?

Si vous habitez en France depuis moins d'un an, indiquez 0

/__/__/ ans

Q26. Le français est-il votre langue maternelle ?

1. Oui
2. Non

Q27. Quel est le diplôme le plus élevé que vous ayez obtenu ?

1. Aucun diplôme
2. Certificat d'études primaires ou diplôme étranger de même niveau
3. CAP, BEP ou diplôme de ce niveau
4. Brevet des collèges, BEPC, brevet élémentaire ou diplôme étranger de même niveau
5. Baccalauréat technologique ou professionnel ou diplôme de ce niveau dont BT et BTA…
6. Baccalauréat général, brevet supérieur, capacité en droit, DAEU, diplôme étranger de ce niveau
7. Baccalauréat sans autre précision
8. Diplôme de niveau bac+2 (DEUG, DUT, BTS)
9. Diplôme de niveau bac+3 ou bac+4 (2nd cycle, master 1, licence ou maîtrise)
10. Diplôme de niveau bac+5 ou supérieur (3ème cycle, master 2, DESS, DEA, diplôme d'ingénieur, de grande école, doctorat…)

Q28. Actuellement, diriez-vous qu'au sein de votre foyer, financièrement…

1. Vous êtes à l'aise
2. Ça va
3. C'est juste, il faut faire attention
4. Vous y arrivez difficilement
5. Vous ne pouvez pas y arriver sans faire de dettes

6. Je ne souhaite pas répondre
